# Nonlinearity in the Mechanical Response of Rubber as Investigated by High-Frequency DMA

**DOI:** 10.3390/polym11040581

**Published:** 2019-04-01

**Authors:** Imran Hussain Syed, Pascal Vouagner, Frank Fleck, Jorge Lacayo-Pineda

**Affiliations:** 1 Continental Reifen Deutschland GmbH, Jaedekamp 30, 30419 Hannover, Germany; Frank.fleck@conti.de (F.F.); jorge.lacayo-pineda@conti.de (J.L.-P.); 2 Metravib, 200 Chemin des Ormeaux, F-69578 Limonest, France; pascal.vouagner@acoemgroup.com

**Keywords:** nonlinearity, superharmonic resonance, foldover effect, HF DMA

## Abstract

Nonlinear material response is analysed with the Fourier transform (FT) of the raw signal measured by a high-frequency dynamic mechanical analyzer (HF DMA). It is known from rheological behaviour of elastomers that reinforcing fillers additionally induce nonlinearity in an already inherently nonlinear system. This behaviour is often described in terms of a mechanical response of strain sweeps, essentially the transition from the linear viscoelastic (LVE) to the nonlinear viscoelastic (NVE) region. In the current investigation, the NVE region could be observed with respect to frequency under low-amplitude deformation. A foldover effect was observed, whereby the material exhibited a nonlinear dependency in relation to the increment of the filler amount above the percolation threshold. In addition, an apparent superharmonic resonance was observed within higher orders of vibrational modes which is further indication of nonlinearity. In this paper, the analytical approach is presented as a novel method to characterise the behaviour of the polymer–filler interaction by HF DMA.

## 1. Introduction

Rubber is one of the most prevalent among the materials in our daily life that exhibit mechanical nonlinearity. Even with this familiarity, deep understanding of this material remains a substantial challenge to this very day. A common way to characterise rubber is with the dynamic mechanical analyzer (DMA). The basic principle behind this technique is the measurement of the difference between the excitation signal and the material’s response as a function of both deformation and time. A material is defined as more viscous (or rubbery) when the difference between these parameters is large [1].

All elastomers have a region whereby the modulus of the system is independent of the strain applied. However, after a certain strain threshold the modulus drops significantly with respect to the applied amplitude. This is mainly due to the rearrangement of the polymer chains with respect to the external applied force, and is also reflected in the stress–strain curve, where the mechanical response portrays a non-Hookean behaviour [2]. The introduction of fillers lowers the critical strain threshold and is caused by a combination of the hydrodynamic reinforcement of the filler particles and the formation of a filler network [3]. The breakdown of this filler network is described as the so-called “Payne effect” [4].

In a routine DMA measurement, a simple linear viscoelastic material is assumed, whereby only the absolute amplitude and phase shift of the signals are taken into consideration. In reality, however, these signals are rarely ideally sinusoidal and are a convolution of both the imperfection derived from the oscillator as well as the nonlinearity of the material. Assuming that the imperfection of the oscillator is minimal, these signal distortions can be used to describe the material in greater detail. This method is known as Fourier transform (FT) rheology and has been developed by various research groups [5,6,7].

Recently, systematic investigations of the influence of fillers in rubber have been performed by Wilhelm et al. whereby the experimental raw signals from amplitude variation up to very large deformations were converted into Fourier space, hence decomposing the distortion into quantifiable signals. These decomposed signals show a strong dependency on the amount and types of reinforcing fillers present in the material [8,9]. The main parameter used to distinguish the filler-induced nonlinearities is the intensity ratio of the third to the first harmonic response in Fourier space, $I_{3/1}$ [10].

In analogy, instead of amplitude variation, the raw data obtained by frequency variation was analysed in Fourier space under low-amplitude deformation. While the strain dependency of nonlinearity has been extensively investigated, the frequency behaviour at low strain amplitude is often assumed to be completely linear, and some clarification is therefore needed. In addition, the current method based on strain sweeps requires uncured rubber, while cured rubber is utilised in the present investigation, as it is the state linked to the final application of rubber products. Henceforth, this paper can be categorised into three sections: the data treatment, where all the analytical steps are shown; the vibrational superharmonics; and the foldover effect. These effects are the consequence of the nonlinearity effects of the material [11].

## 2. Materials and Methods

**Figure 1:**
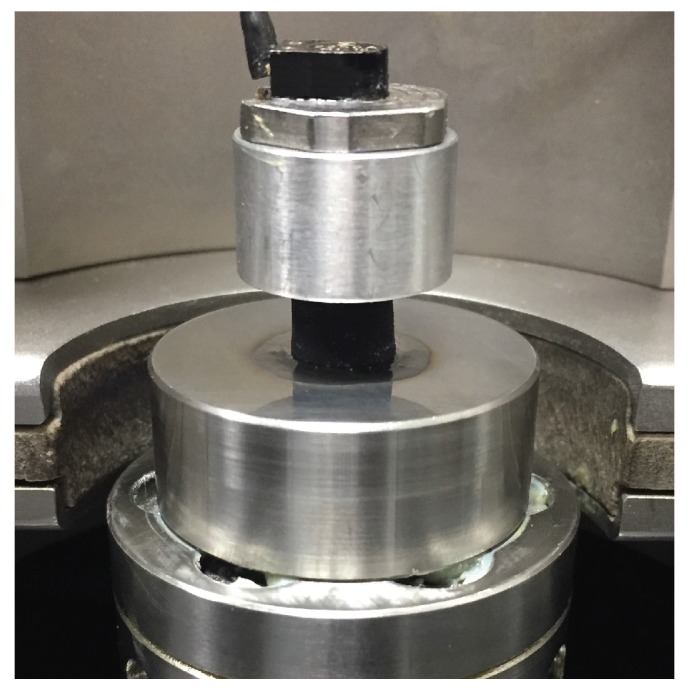
The experimental setup for the VHF104 dynamic mechanical analyzer.

**Figure 2:**
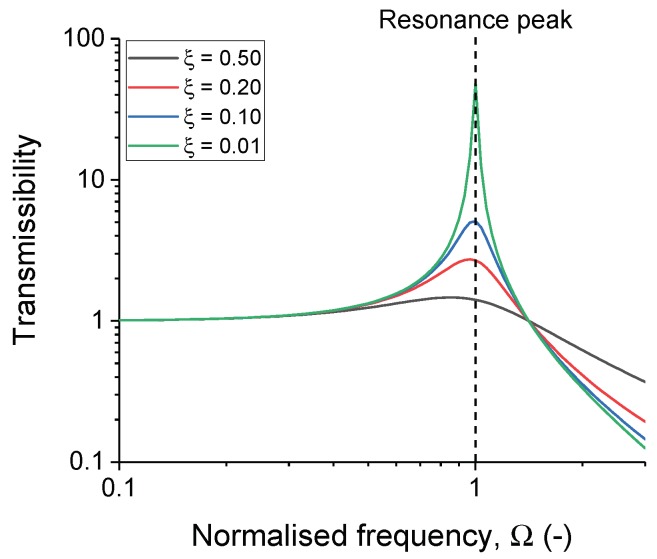
An example of the transmissibility curve generated from Equation (1), with various damping factors.

A VHF104 high-frequency DMA (Metravib, Lyon, France), as shown in Figure 1, was used in the current investigation. The sample was glued between two metal cylinders, one acting as a base and the other to deliver a preload force onto the sample. The main advantage of this device is the ability to measure frequencies up to 10 kHz, which is not possible with a traditional DMA device (typically up to 100 Hz). This DMA is a type of forced vibration resonant system, according to ASTM D5992 [12], essentially using the resonance principle to evaluate the mechanical response of the material. The data are presented in a transmissibility plot as shown in Figure 2. The figure was generated by using the transmissibility formula in Equation (1), where ξ is the damping ratio of the system and Ω is the resonance-normalised frequency of the system:

(1) Transmissibility=FtransmittedFexcited=1+2·ξ·Ω(21−Ω(2+2·ξ·Ω(2.

**Figure 3:**
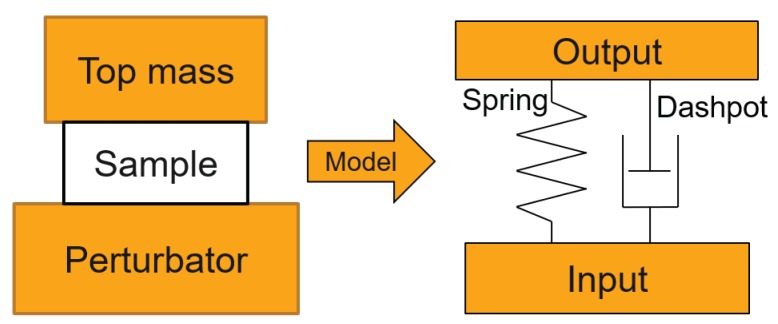
Basic schematic for the current experimental setup.

The experimental setup was essentially comprised of two components: the base excitation input and the top transmission output, as illustrated in Figure 3. The viscoelastic properties of a material can be approximated in terms of a spring (elastic component) and a dashpot (viscous component) [13]. The base excitation generates a defined, constant excitation that passes through the sample, and the transmitted signal is received on the accelerometer located at the head of the top mass.

Resonance occurs when a perturbation frequency matches a system with its natural frequency. Under resonance, the system is able to exhibit dynamics with a larger amplitude than the initial perturbation state. The transmissibility curve, which is a measure of the maximum amplitude response normalised to the maximum amplitude of the input, is one of many ways to represent resonance-based data. The abscissa is often normalised to the natural frequency, which means that the resonance peak occurs when the normalised frequency is equal to 1. In theory, the transmissibility of the resonance peak should increase asymptotically, but is limited by the damping behaviour of the material. In the current investigation, the transmissibility curve will be further investigated with Fourier analysis. [14]

Since this method uses the resonance phenomenon, the experimental setup must be calibrated in terms of sample geometry and added mass, in order to obtain the material resonance in the desired frequency window. The resonance frequency $f_{o}$ in tension–compression mode can be estimated with the following equation:

(2) $$f_{o}=\frac{1}{2\pi }\sqrt{\frac{E\cdot S}{l\cdot M}},$$

where *E* is the Young’s modulus in N/m${}^{2}$, *S* is the cross-sectional contact area in m${}^{2}$, *l* is the height of the sample in m and *M* is the added top mass in kg.

The temperature range of operation for the VHF104 is given from −50 to $100{\;}^{\circ }$C, however in the present work, only ambient conditions were considered for simplicity. In terms of excitation, the transducer was able to perturbate up to 200 m/s${}^{2}$. The deformation amplitude was operating in the micrometer range in order to probe the strain-dependent linear viscoelastic region of the rubber sample. In the current setup, tension–compression mode was used due to the geometric simplicity. While it is known that this deformation mode induces nonlinearity due to geometrical effects, the current setup used a small deformation amplitude, hence minimising the aforementioned effect. In addition, the sample diameter (8 mm) was sufficiently larger than the height (6 mm) of the sample, therefore reducing the risk of transverse bending with respect to the deformation axis.

The material chosen for the investigation was a simple solution-polymerised styrene-butadiene rubber (SSBR) compound with various amounts of carbon black N339 fillers, and is summarised in Table 1.

## 3. Results and Discussion

### 3.1. Data Processing

Analog acceleration-time signals were sampled and directly transformed into frequency data using synchronous detection algorithms. For this device, up to three harmonics were observed with significant signal-to-noise ratio with several orders of magnitude. For simplicity, the raw signals will be denoted as input and output signal.

Both the input and output signals were extracted via the built-in option of the standard evaluation software, Dynatest, made for VHF104. The resultant file from each measured frequency $f_{expt}$ contained 512 time-independent points of acceleration amplitude denoted by the incremental value of *n*, hence a time variable $t_{n}$ was assigned with the known frequency:

(3) $$t_{n}=\left(\frac{n}{512}\right)\left(\frac{1}{f_{expt}}\right).$$

**Figure 4:**
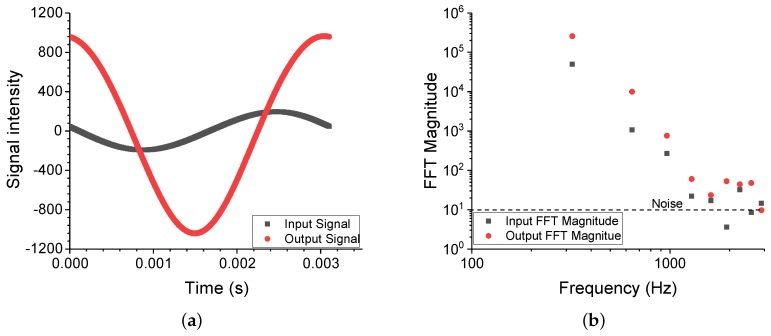
(**a**) Time-domain signal of a 10 phr filled SSBR sample at the resonance frequency of 300 Hz and (**b**) the corresponding Fourier space of the signal. Note that the first three harmonics were several orders of magnitude higher than the background noise.

These time-domain signals were then transformed into Fourier space, and the result of this procedure is shown in Figure 4. This procedure obeys all three fundamental Fast Fourier Transform (FFT) prerequisites, and therefore the analysis is representative of the real data [14]. The first prerequisite is that the signal was periodic and continuous. Secondly, the sample size was sufficiently large to be representative of the real signal. Finally, the spectral leakage of the signal was minimised by taking values of $2^{n}$. The lowest frequency point (i.e., the first data point) in Figure 4b indicates the first harmonic of the system, which is essentially the main signal used to characterise the viscoelastic behaviour of the material. Apart from spectral leakage, whereby the signal is superficially distorted due to sampling issues from the Fourier transformation, a single peak is expected from a linear or homogeneous system. Evidently, this was not the case as higher harmonics were observed, provided that the frequency range did not exceed the VHF104 excitation limitation of 10 kHz. It should be noted, however, that since the input waveform becomes more erratic as the harmonics increase, only the first three harmonics should be considered for further analysis.

Since the output signal contained residues from the input, normalising the signal gave the theoretical pure signal of the sample. Performing this on each experimental point produced a transmissibility or transfer function curve when plotted against frequency.

(4) $$Transmissibility=\frac{\mathrm{Output}\mathrm{signal}}{\mathrm{Input}\mathrm{signal}}$$

**Figure 5:**
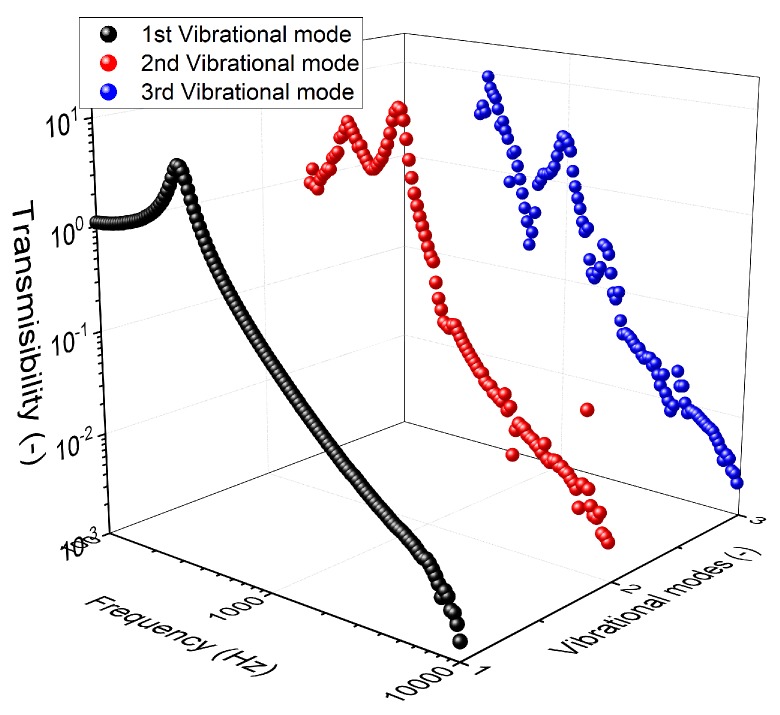
The transmissibility (output normalised to input) curve of the first three vibrational modes.

The final outcome of the process is shown in Figure 5. The vibrational modes were defined as the transmissibility of the vibrational harmonics. For example, the intensity-normalised second harmonic of a sample measurement was denoted as the second vibrational mode. Interestingly, all the vibrational modes coincided with one another at certain frequency ranges, which indicates that the first vibrational mode was affecting the subsequent higher-order vibrational modes. Note that the higher vibrational modes were multiple integers of the first vibrational mode. Further discussion will follow in the next section.

### 3.2. Vibrational Superharmonic

The term *vibrational superharmonic resonances* was first quoted by Yang et al. [15] in order to describe the appearance of higher-order resonances seen in nonlinear systems. Their investigation is mostly focused on the frequency response of a bistable system, whereby the system is perturbated with two excitation signals, one being a significantly lower frequency than the other. Nevertheless, it was noted that a high-frequency perturbation on a nonlinear system can induce both vibrational superharmonic resonance and vibrational subharmonic resonance.

The existence of higher vibrational modes was a unique trait from this experimental setup and could lead to hints on the nonlinearity of the system. In order to probe these higher vibrational modes, a baseline has to be defined as to remove the influence of the prior vibrational mode. In the VHF104, a harmonic vibration was applied at the base of the sample and the so-called reaction mass was bonded at the top of the sample. Assuming a linear system, the equation of motion where the first three terms are defined as the inertia, damping and elastic components of the system is defined as:

(5) $$m\ddot{x}+c\left(\dot{x}-{\dot{x}}_{e}\right)+k\left(x-x_{excitation}\right)=0.$$

Equation (5) can be further simplified by combining the excitation terms and defining them as an equivalent excitation force, $F_{e}cos\left(\omega t\right)$ with ω=2πf, the pulsation of the applied vibration:

(6) $$m\ddot{x}+c\dot{x}+kx=F_{e}cos\left(\omega t\right).$$

The resultant transmissibility is given in Equation (7) [16], where *f* is the measured frequency, $f_{o}=\sqrt{\left(k/m\right)}/2\pi$ is the natural frequency of the dynamical system composed of the sample and the reaction mass, and $\xi =c/c_{c}$ is the damping ratio of the system—essentially the percentage of measured damping with respect to the critical damping, $c_{c}=2\sqrt{km}$. As a first approximation, this equation was able to fit the first vibrational mode and was therefore used as a baseline for the subsequent steps.

(7) |TLinear|=FtransmittedFexcited=1+2·ξffo(21−f2fo2(2+2·ξffo(2

**Figure 6:**
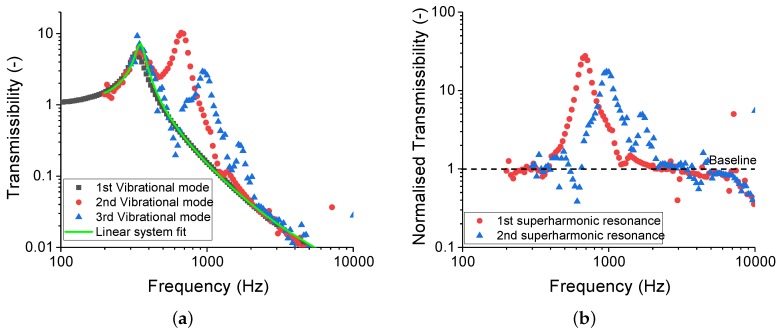
The transmissibility curve for SSBR10CB (**a**) before and (**b**) after normalising to the fitted linear vibration equation. The first superharmonic resonance was defined as the resonance of normalised second vibrational mode.

**Figure 7:**
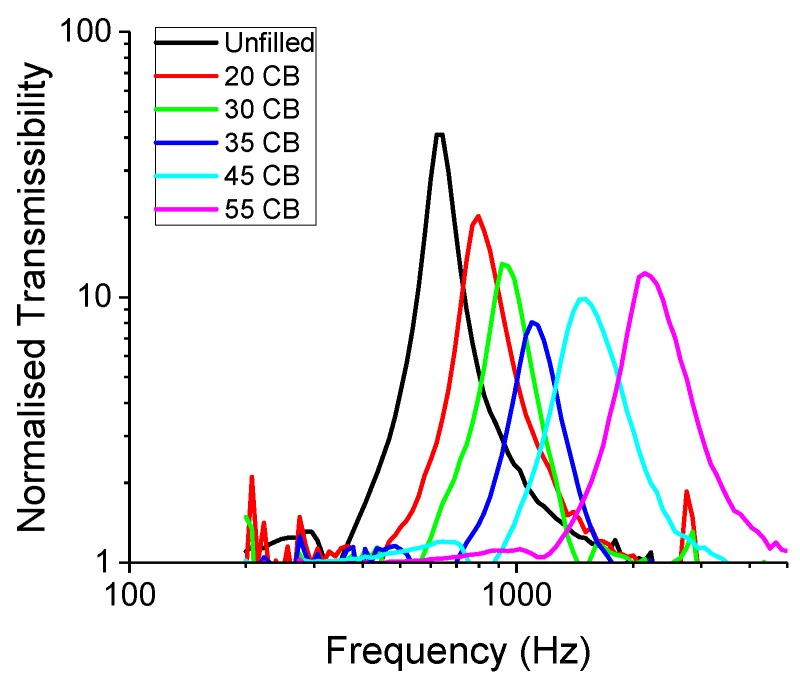
The normalised second vibrational mode as a function of frequency for selected filler loading.

Normalising the second vibrational mode to the fitted first vibrational mode led to a pure signal response of the mode, as shown in Figure 6. A similar procedure can be done for the third vibrational mode. At this point, the maximum of the normalised vibrational modes were defined as the superharmonic resonances. The normalised second vibrational mode for the selected filler loading is shown in Figure 7.

**Figure 8:**
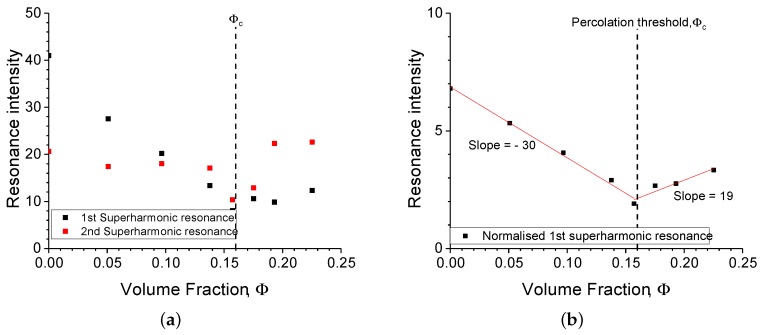
(**a**) The intensity of the first and second superharmonic resonance and (**b**) the intensity of the first superharmonic resonance normalised to the maximum of the first vibrational mode, $I_{2/1}$.

Taking the amplitude of the superharmonic resonances for all measured samples, the results are plotted in Figure 8a. The second superharmonic resonance had a lower intensity and was more prone to noise as seen in Figure 4b. Therefore, the first superharmonic resonance was the focus of the current investigation. Interestingly, the amplitude of the first superharmonic resonance was a function of the filler amount present in the sample. Its amplitude was reduced with increasing amount of filler, up to the point where the filler percolation threshold $\phi_{c}$ occurred. This seems to indicate that the filler was suppressing the intrinsically nonlinear behaviour of the elastomer, up to a point where a filler network was formed ($\phi_{c}$≈ 0.16 for N339). Beyond this point, the system exhibited a relatively weak nonlinear behaviour, which could be attributed to the aggregation of the filler network. This observation is similar to the maxima observed in elongation at break with the variation of filler amount [17] . At this point, a conclusive statement requires a more thorough investigation as well as the consideration of other possible methods, such as normalising the higher harmonic with the first (see Figure 8b). Such is the case for the Fourier analysis of the rheological test in large-amplitude oscillatory shear (LAOS), whereby the third harmonic is normalised to the first ($I_{3/1}$) [8].

### 3.3. Foldover Effect

Another consequence of the nonlinear resonance response is the foldover effect, whereby the transmissibility of the resonance is seen to be more asymmetric with respect to the frequency and amplitude of the oscillation. One of the earliest mathematical descriptions of the nonlinear resonance response was made by Landau and Lifschitz, whereby they assumed that the nonlinearity could be described as higher-order terms of the viscous and elastic components of a system [11]. In the present work, only the additional third-order spring term was considered as a first approximation of the foldover effect. Therefore, it was assumed to be a double-potential well system, which is more physically relevant [18]—that is, there was an energetic minimum for the integral of nonlinear spring, $x+x^{3}$. As a first approximation, the emphasis was placed on the influence of the nonlinearity of the elastic component to characterise the asymmetry of the resonance peak, in analogy to the previous observation in micro-electro-mechanical systems (MEMS) [19].

**Figure 9:**
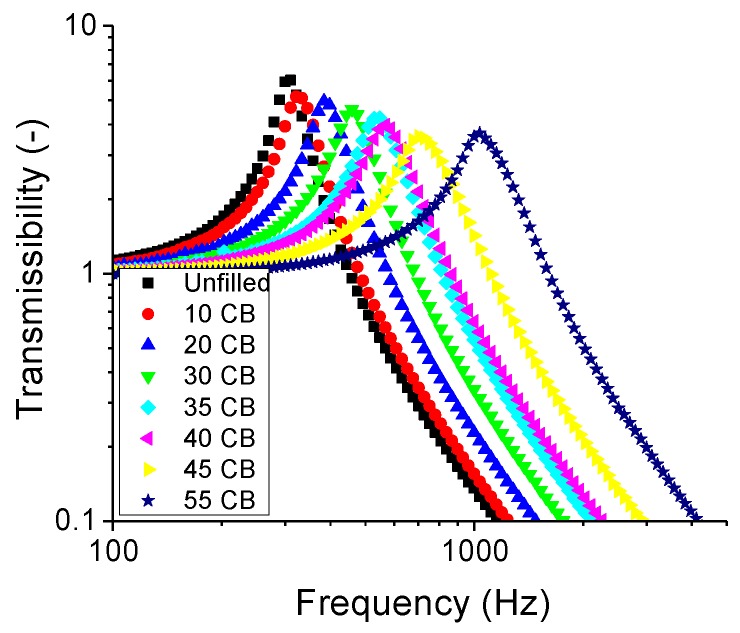
The transmissibility curve of the first vibrational mode with N339 CB.

The first vibrational mode is presented in Figure 9. It can be clearly seen that as the filler amount increased, the resonance peak got progressively more asymmetric. One possible explanation here is that the mechanical response of the filler was vastly different from that of the rubber matrix. Hence, the filler network was not easily deformable as the rubber matrix counterpart. A simple nonlinear response model for a forced oscillation is given in Equation (8). This equation is also known as the Duffing’s equation [18].

(8) $$m\ddot{x}+c\dot{x}+k_{1}x+k_{2}x^{3}=F_{e}cos\left(\omega t\right)$$

The consequence of this equation in terms of transmissibility is given by Equation (9) [20], where $\hat{X}$ is the amplitude of the output signal, α is the cubic nonlinear coefficient, ξ is the damping ratio and Ω (=$f_{expt}/f_{resonance}$) is the resonance-normalised frequency:

(9) $$|T_{Nonlinear}|=\hat{X}\sqrt{{\left(1+\frac{3}{4}\alpha {\hat{X}}^{2}\right)}^{2}+4\xi^{2}\Omega^{2}}.$$

**Figure 10:**
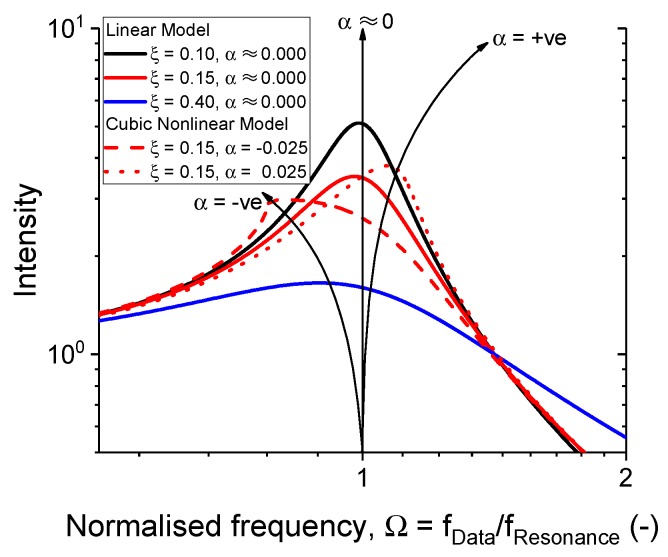
The influence of the cubic nonlinearity coefficient, α-parameter, and the damping factor on the transmissibility curve generated from Equation (9).

Using the aforementioned formula, the degree of nonlinearity of the material response can be quantified with the α parameter. The influence of the α parameter and the damping factor is depicted in Figure 10. For the linear example, the α parameter was set to a very small value to avoid an undefined solution. Comparing Equations (7) and (9) with a small α parameter values led to the same solution.

**Figure 11:**
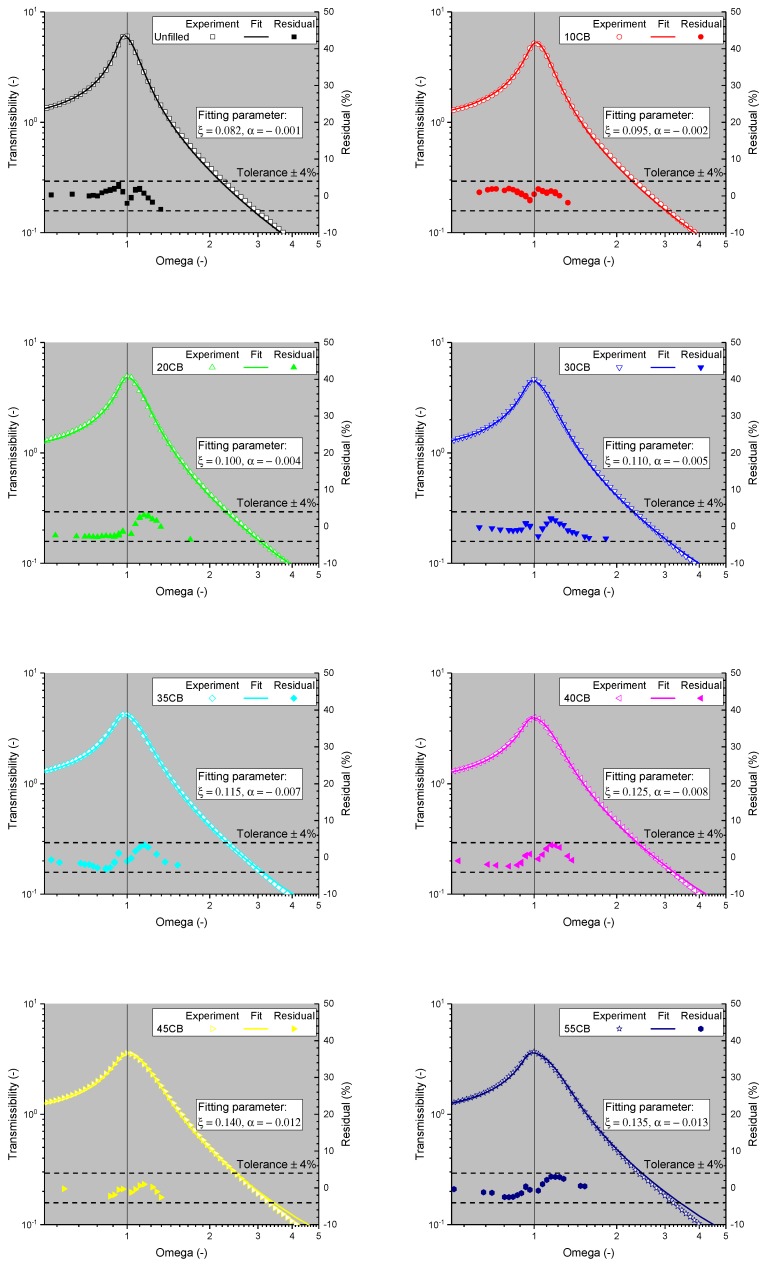
The cubic nonlinearity fit with the corresponding residual values for the current investigation.

The outcome of the fitting procedure is shown in Figure 11. All of the samples had a fitting tolerance of 4%, which was calculated from the residual (=$|\left(T_{experiment}-T_{fit}\right)|/T_{experiment}$). The magnitude of the α parameter was observed to increase with higher CB amounts. This means that the filler induced the higher elastic term, and was therefore making the system more nonlinear. Note that only a negative α parameter value was observed, which indicates a softening behaviour. In micro-electro-mechanical systems (MEMS), a similar cubic nonlinearity is also observed but with a positive α parameter, which is referred to as a hardening phenomenon [19].

**Figure 12:**
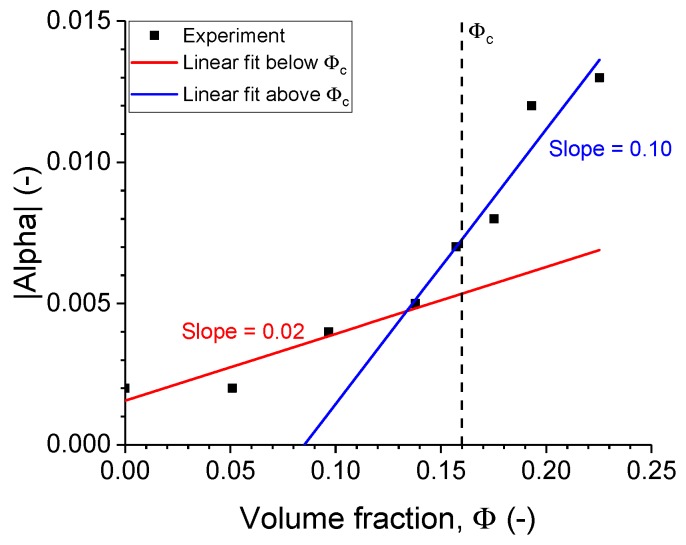
The nonlinear cubic coefficient values for the current investigation. The percolation threshold, $\phi_{c}$, is the value obtained from Figure 8.

The influence of filler amount on the α-parameter values is shown in Figure 12. In contrast to the behaviour of the superharmonic resonance, the α parameter was negligible below the percolation threshold. Its apparent small increase below the percolation threshold was not significant, as it was subjected to the low sensitivity of the first vibrational mode. The α parameter below percolation is to be considered as a numerical artefact obtained by fitting. The increase of the α-parameter was more pronounced beyond the percolation threshold and was in line with the previously observed behaviour of the superharmonic resonance.

Hence, it does seem that the analysis of the superharmonic resonance was the appropriate method of evaluating the nonlinearity since it was able to cover the complete range of filler loading, below and above percolation. While the α parameter is only a fitting parameter for the bending of the transmissibility curve, the superharmonic resonance is a value measured with an excellent signal-to-noise ratio.

## 4. Conclusions

The nonlinear material response induced by the nanocomposite fillers was analysed by a high-frequency DMA. Firstly, the basis of the device was introduced, as well as the analytical steps for the experimental signal. By transforming the signal into Fourier space, several higher harmonics were observed. Normalising these harmonics with the respective input signal led to vibrational modes that contained superharmonic resonances, which are essentially a harmonic oscillation generated by the nonlinearity of the resonance peak. An apparent correlation was observed between the first superharmonic resonance and the filler amounts, which is a strong indication of filler induced nonlinearity since there seemed to be a crossover point at the percolation threshold $\phi_{c}$. In fact, the resonance peak beyond the percolation threshold exhibited a foldover effect, whereby the resonance peak displayed an asymmetrical behaviour. This behaviour could be described with a nonlinear cubic model with an α parameter that denotes the nonlinearity term. This model, however, is limited to filled compounds which are above the percolation threshold, whereby the filler-induced nonlinearity is dominant. Therefore, the superharmonic resonance seemed to be more sensitive, as both the intrinsic nonlinearity of the elastomer and the filler-induced nonlinearity could be observed. Hence, it is more appropriate to evaluate the nonlinearity based on the superharmonic resonance. Future investigations will involve different filler grades and polymer types so as to give a more universal statement on the current findings.

References

## Figures and Tables

**Table 1:** Recipe series used in this study.

Ingredients	Amount (phr) *
SSBR ${}^{a}$	100.0
N339	0.0–55.0
IPPD ${}^{b}$	1.5
Zinc Oxide	3.0
Stearic acid	1.0
CBS ${}^{c}$	2.0
Sulphur	1.4

* Parts per hundred rubber; ${}^{a}$ solution-polymerised styrene-butadiene rubber—styrene content 24%, vinyl content 34%, molecular weight 472.65 kg/mol, Tg $-40{\;}^{\circ }$C, PDI 1.65; ${}^{b}$ *N*-Isopropyl-*N*${}^{\prime }$-phenyl-1,4- phenylenediamine; ${}^{c}$ *N*,*N*${}^{\prime }$-Dicyclohexyl-2-benzothiazole sulfenamide.
